# Research Topic: Measurable Residual Disease in Hematologic Malignancies. Can digital droplet PCR improve measurable residual disease monitoring in chronic lymphoid malignancies?

**DOI:** 10.3389/fonc.2023.1152467

**Published:** 2023-03-14

**Authors:** Giovanni Manfredi Assanto, Ilaria Del Giudice, Irene Della Starza, Roberta Soscia, Marzia Cavalli, Mattia Cola, Vittorio Bellomarino, Mariangela Di Trani, Anna Guarini, Robin Foà

**Affiliations:** ^1^ Hematology, Department of Translational and Precision Medicine, Sapienza University, Rome, Italy; ^2^ Gruppo Italiano Malattie Ematologiche dell'Adulto (GIMEMA), Fondazione GIMEMA Franco Mandelli Onlus, Rome, Italy; ^3^ Department of Molecular Medicine, Sapienza University, Rome, Italy

**Keywords:** digital droplet PCR, measurable residual disease (MRD), non-Hodgkin lymphoma, chronic lymphocytic leukemia, hairy cell leukaemia (HCL)

## Abstract

Minimal/measurable residual disease (MRD) monitoring is progressively changing the management of hematologic malignancies. The possibility of detecting the persistence/reappearance of disease in patients in apparent clinical remission offers a refined risk stratification and a treatment decision making tool. Several molecular techniques are employed to monitor MRD, from conventional real-time quantitative polymerase chain reaction (RQ-PCR) to next generation sequencing and digital droplet PCR (ddPCR), in different tissues or compartments through the detection of fusion genes, immunoglobulin and T-cell receptor gene rearrangements or disease-specific mutations. RQ-PCR is still the gold standard for MRD analysis despite some limitations. ddPCR, considered the third-generation PCR, yields a direct, absolute, and accurate detection and quantification of low-abundance nucleic acids. In the setting of MRD monitoring it carries the major advantage of not requiring a reference standard curve built with the diagnostic sample dilution and of allowing to reduce the number of samples below the quantitative range. At present, the broad use of ddPCR to monitor MRD in the clinical practice is limited by the lack of international guidelines. Its application within clinical trials is nonetheless progressively growing both in acute lymphoblastic leukemia as well as in chronic lymphocytic leukemia and non-Hodgkin lymphomas. The aim of this review is to summarize the accumulating data on the use of ddPCR for MRD monitoring in chronic lymphoid malignancies and to highlight how this new technique is likely to enter into the clinical practice.

## 1 Introduction

Monitoring of measurable/minimal residual disease (MRD) is progressively impacting on the management and outcome of different hematologic malignancies, since it can predict patients’ outcome, redefine prognostic risk stratification and response to treatment and in acute leukemias and chronic myeloid leukemia also guide treatment decisions (1–5). Several molecular techniques are employed to monitor MRD, from conventional real-time quantitative polymerase chain reaction (RQ-PCR) (6–9) to next-generation sequencing (NGS) (10–13) and digital droplet PCR (ddPCR) (14–17), through the detection of fusion genes, immunoglobulin (IGH) or T-cell receptor (TCR) gene rearrangements, or disease-specific mutations. They are applied to different tissues or compartments, i.e. bone marrow (BM) and peripheral blood (PB) - for both genomic DNA from circulating neoplastic cells or circulating cell-free DNA (cfDNA) from plasma (18, 19).

RQ-PCR still represents the gold standard for MRD. International guidelines for analysis and reporting have been established by the EuroMRD Consortium (8). Despite the high sensitivity of RQ-PCR, a non-negligible fraction of samples with low-level positivity within the 1 x 10^-4^ to 1 x 10^-5^ range (i.e. 1 tumor cell within 10.000-100.000 normal cells) cannot be precisely quantified according to the EuroMRD guidelines (20). The reason could reside in the lack of reproducibility of the samples at these levels. However, in most cases it is difficult to distinguish the PCR amplification signal of very few residual leukemic cells from the non-specific signal (20). Moreover, MRD quantification by RQ-PCR is based on a standard curve built on the dilution of the diagnostic sample within a pool of healthy donors’ DNA.

NGS, widely employed to detect disease-specific mutations with high sensitivity (<1%) when compared to Sanger sequencing (10-20%), can also be employed for target screening and MRD monitoring. It shows the remarkable advantage of a wide applicability (≥95% of cases) and of providing additional information on the whole clonal composition and/or clonal evolution of each neoplasm. The EuroMRD Consortium has recently established the indications to apply NGS for target screening (10–13). However, since NGS sensitivity for MRD detection increases with the increase of DNA input, the issue of the balance between costs and feasibility is still a matter of debate.

ddPCR, considered the third-generation PCR, yields a direct, absolute, and accurate detection and quantification of low-abundance nucleic acids, with documented advantages in the context of MRD quantification (see below). ddPCR is actively investigated in the context of the EuroMRD group. At present, standard operating procedures have been published as a guide for digital analysis in lymphoid malignancies (21).

NGS and ddPCR could also be applied in combination: NGS can be optimized to detect the target sequence of IGH rearrangements, which can be employed to design patient-specific probes to be monitored by ddPCR, which allows to reduce costs, time and efforts compared to NGS monitoring.

At present, the use of ddPCR and NGS to monitor MRD in the clinical practice is limited by the lack of international guidelines. Nevertheless, their application within clinical trials is progressively growing in lymphoid malignancies, such as Philadelphia-positive and -negative acute lymphoblastic leukemia (ALL), chronic lymphocytic leukemia (CLL) and non-Hodgkin lymphomas (NHL) (1–4, 15–17, 20, 22).

The aim of this review is to summarize the accumulating data on the use of ddPCR for MRD monitoring in chronic lymphoid malignancies and to highlight how this new technique can enter into the clinical practice.

## 2 Technical principles of ddPCR

The ddPCR system is based on the generation of droplets through a water-oil emulsion of the sample. This partitioning process allows to obtain multiple PCR sub-reactions, in which each generated droplet contains single, few or no target sequences (23, 24). PCR partitions are read and counted as negative or positive by thresholding based on their fluorescence amplitude. Based on Poisson’ statistics, the number of positive and negative partitions is used to calculate the concentration of the target sequence, which can be a known mutation or a “patient-tailored” sequence (25, 26).

High precision and sensitivity (down to a level of detection of 0.001%) are given by compartmentalization that renders PCR less sensitive to reaction inhibitors, and reduces any template competition, allowing the detection of rare target sequences in a wild-type background (26–29). Assays are evaluated on the basis of specific parameters: Limit of Blank, which is the highest amplitude in which a blank sample stands when it is not containing any target sequence; Limit of Detection, the lowest amplitude at which target amplification can be distinguished from the blank; Limit of Quantification, the lowest concentration at which a target sequence can be quantified (25, 29). However, ddPCR still requires a marker-specific tuning of PCR reactions, i.e. annealing temperature, primer/probes concentration and, for results analysis, a manual positioning of a threshold cycle. In addition, at variance from NGS, ddPCR has technical limitations in the multiplex approach.

In the MRD setting, while RQ-PCR quantification is relative to a standard curve built on the dilution of the diagnostic sample in a pool of DNA from healthy donors, ddPCR MRD evaluation is an absolute quantification that makes unnecessary the standardized dilution curve at each time point of disease monitoring. Adaptability, reproducibility and ease of use are distinctive features of this method, that has spread in the general practice.

## 3 ddPCR in chronic lymphoid malignancies

With the advent of chemo-immunotherapy and, more recently, with the introduction of new targeted agents in various combinations, the prognosis of CLL and NHLs has considerably changed over the years. Complete responses are increasing in rate and long-lasting over time. However, a consistent proportion of patients experiences a relapse after achieving a complete remission. Thus, MRD analysis has acquired relevance in the effort of predicting patients’ outcome, stratifying more accurately patients into risk categories, redefining the clinical response to treatment, and possibly optimizing treatment strategies also in chronic lymphoid malignancies (2–4, 16).

During the last few years, ddPCR has been investigated for the monitoring *BCL2::IGH* rearrangement in follicular lymphoma (FL), *BCL1::IGH* in mantle cell lymphoma (MCL), *MYD88* mutations in Waldenstrom macroglobulinemia (WM) and *IGH* rearrangements in chronic lymphocytic leukemia (CLL), proving a promising tool to further refine MRD monitoring **(** **Table 1** **).**

**Table 1:** Experiences reporting on ddPCR and MRD in lymphoproliferative disorders.

Studies comparing ddPCR to RQ-PCR							
Study	Disease	N° of patients (samples)	Rationale	Tissue Timing	Marker	Concordance with RQ-PCR	Major Advantages of ddPCR
Drandi et al.^28^	FL+MM+MCL	30+18+21 (222)	Comparison between RQ-PCR and ddPCR	BM at diagnosis and MRD	*BCL2::IGH* *IGH*	85%	7 of 26 PNQ samples (26.9%; five MM, one MCL, and one FL) by RQ-PCR were quantified by ddPCR; 6/26 (23.1%) were negative by ddPCR
Cavalli et al.^27^	Early stage FL	67 (138)	Comparison between RQ-PCR and ddPCR	PB+BM at diagnosis and MRD	*BCL2::IGH*	81.9%	8/18 (44.4%) negative at diagnosis were MBR+ by ddPCR Tumor burden at diagnosis correlates with PFS only when quantified by ddPCR
Drandi et al.^29^	MCL	166 (416)	Comparison between RQ-PCR and ddPCR	PB+BM at MRD	*BCL1::IGH* *IGH*	ICC = 0.79, 95% CI: 0.75-0.83	Among 240 PNQ samples at qPCR, 39% were positive by ddPCR, 49% negative and only 12% remained positive below quantifiable ddPCR limits
Drandi et al.^46^	WM	148 (291)	Reliability of ddPCR to detect *MYD88* ^L265P^	PB+BM ctDNA at diagnosis	*MYD88* ^L265P^	/	122 of 128 (95.3%) BM and 47/66 (71.2%) baseline PB samples scored positive for MYD88L265P. High concordance between ctDNA and BM levels
Della Starza et al.^75^	ALL, CLL, MCL, FL	216 (620)	Comparison between RQ-PCR and ddPCR	PB+BM at diagnosis and MRD	IGH TCR BCL2::IGH	76.4%	Significant reduction of PNQ samples, from 18% to 11% Significant increase of quantifiable MRD, from 29% to 38.4%
Guerrini et al.^81^	HCL, SMZL	47 (141)	Comparison between RQ-PCR and ddPCR	BM+PB at diagnosis and MRD	BRAF V600E	/	Sensitivity of ddPCR is about half a logarithm superior to RQ-PCR Superiority in the identification of MRD+ after treatment
Clinical trials employing ddPCR for MRD monitoring							
Study	Disease	N° of patients	Therapy	Tissue Timing	Marker+ at diagnosis	MRD- at EOI	Clinical impact
Delfau-Larue et al.^42^	Untreated advanced FL	440	Phase 3 Relevance trial. Rituximab plus lenalidomide (R2) vs R-CHOP, both arms were followed by rituximab maintenance	PB+/-BM at diagnosis and MRD	222/440 (50.45%) BCL2::IGH+	MRD- at EOI (week 24): PB 98% and BM 78% R2 arm: MRD- 90% (105/117) R-CHOP arm: MRD- 77% (70/90)	3-Year PFS: 84% for MRD- vs 55% for MRD+ 3-Year PFS: 85% for BM MRD- vs 54% for BM MRD+ MRD+ at EOI: HR 3.3 (1.2-9.2, p=.02) for R-CHOP arm HR 2 (0.6-6.8; p=.27) for R2 arm
Pulsoni et al.^36^	Untreated localized FL Stage I (78%)- Stage II (22%)	67	IFRT (24-30Gy) + 4 weeks of Rituximab in MRD+	PB+ BM at diagnosis and MRD	72% BCL2::IGH+	MRD- after RT:50% MRD- after R:84% In MRD+ post IFRT: superior PFS in patients treated with R vs untreated with R	84-m PFS: 75% for BCL2/IGH- vs 59% for BCL2/IGH+ by RQ-PCR at baseline (p=.26) 84-m PFS: 90.9% in 11 pts with MRD <10^-5^ vs 38% in 19 pts with MRD_=_10^-5^ by ddPCR at baseline (p=.015)

It includes studies comparing ddPCR to RQ-PCR for MRD monitoring or clinical trials with ddPCR-based MRD. ddPCR, digital droplet polymerase chain reaction; RQ-PCR, real quantitative polymerase chain reaction; ALL, acute lymphoblastic leukemia; FL, follicular lymphoma; HCL, hairy cell leukemia; MCL, mantle cell lymphoma; MM, multiple myeloma; SMZL, splenic marginal zone lymphoma; WM, Waldenstrom macroglobulinemia; BM, bone marrow; PB, peripheral blood; ctDNA, circulating tumor DNA; PNQ, positive not-quantifiable; MRD, minimal residual disease; IFRT, involved field radiotherapy; ICC, intraclass correlation; EOI, end of induction; PFS, progression-free survival.

### 3.1 Follicular lymphoma

The genetic hallmark of FL is the *BCL2::IGH* rearrangement, which is a result of the t (14, 18) (q32;q21) translocation which enhances anti-apoptotic activity posing the *BCL2* gene under the transcriptional control of the heavy chain gene enhancer. The rearrangement can occur in the major breakpoint region (*MBR*) or, rarely, in the minor cluster region (*mcr*) (30, 31). It is detectable at diagnosis by conventional PCR in 50-60% of cases with advanced FL both by qualitative and quantitative approach (31–35). This low sensitivity can be explained by the employment of large internal primers which target both chromosomes 14 and 18 in the qualitative reaction and the proximity of breakpoints site to target sequences for RQ-PCR (31). In localized FL, the *BCL2::IGH* rearrangement is found in a lower proportion of cases, especially when staged by PET/CT in comparison with historical series (36).

MRD in FL is of great potential value given the heterogeneous clinical behavior of the disease. Large clinical trials in the last years have tried to validate MRD assessment in FL through BCL2::IGH monitoring (32–35). MRD negativity is predictive of a better progression-free survival (PFS) in all clinical trials conducted in the past two decades, even in relapsed patients, and possibly of a longer survival in studies with a prolonged follow-up (4). Nonetheless, MRD monitoring is to date not included in the recommended guidelines for FL management (37).

The introduction of chemo-immunotherapy with anti-CD20 monoclonal antibodies has allowed an increase in the rates of MRD negativity at the end of induction (EOI) up to 70-80% (rituximab-based) and 90% (obinutuzumab-based), respectively (4). Anti-CD20 maintenance holds and increases the rates of MRD negativity. Recently, the assessment of MRD at earlier time points with respect to EOI has been tested for the first time in the Gallium trial and has proven informative (38, 39).

MRD analysis is also a sensitive tool to refine clinical response assessment in FL. The combination of molecular and metabolic-defined response is a promising and valuable tool to be further explored, as well as the possibility of a MRD-driven modulation of the post-induction therapy in FL (35).

Given this landscape, it is clear which clinical benefit could come from optimizing the use of ddPCR in FL to maximize the sensitivity of *BCL2/IGH* detection. The droplets are analyzed on the basis of FAM fluorescence BCL2/JH-linked and corrected by the unspecific background fluorescence. *BCL2::IGH* can be detected down to 1 × 10^−4^ *BCL2/JH*-positive cell line (limit of detection).

Drandi et al. (28) compared RQ-PCR to ddPCR in 30 patients with FL, 18 with multiple myeloma (MM) and 21 with MCL. A highly significant level of concordance was observed between qPCR and ddPCR (r = 0.94, P <0.0001; 95% CI, 0.94–0.97), with 189 of 222 samples (85.1%) fully concordant. In the MRD quantification of 26 samples resulting positive not-quantifiable (PNQ) by RQ-PCR, 27% resulted quantifiable and 23% negative when assessed by ddPCR. This experience showed how ddPCR can be a valid option for MRD detection.

Cavalli et al. (27) tested a cohort of 67 patients affected by early-stage FL both in the PB and BM at diagnosis and after radio-immunotherapy. Among 138 samples, the concordance between RQ-PCR and ddPCR was 81.9%, which raised to 97.5% for the subset with quantifiable disease (40/138) (21). Moreover, at baseline ddPCR identified a MBR marker in 8 of 18 (44%) samples that by qualitative nested PCR resulted as MBR−/mcr−. A molecular tumor burden at diagnosis ≥1 x 10^−5^ significantly predicted PFS only when quantified by ddPCR but not by RQ-PCR (36). Again, a higher sensitivity of ddPCR was shown in RQ-PCR PNQ samples (27).

Della Starza et al. (40), through a collaborative effort of four laboratories belonging to the Fondazione Italiana Linfomi (FIL) MRD Network for FL and MCL MRD assessment, demonstrated that there is a proportion of “borderline” samples (31/187, 17%), those resulting alternatively positive and negative by RQ-PCR/qualitative PCR, that challenge the inter-laboratory reproducibility. There was no inter-laboratory discordance when “borderline” samples were tested by ddPCR analysis.

In another experience by Delfau-Larue et al. (41) quantification of circulating *BCL2/IGH*+ cells and cfDNA was retrospectively performed by ddPCR in 133 FL patients. PB was tested for *BCL2::IGH* rearrangement and the *ANKRD30B* gene was used as the reference gene to quantify the cell-free circulating equivalent genome using the PrimePCR ddPCR copy number assay. A significant correlation was found between the total metabolic tumor volume (TMTV) and both circulating tumor cells (CTCs) (*P <*0.0001) and cfDNA (*P* <.0001). With a median follow-up of 48-month, the 4-year PFS was lower in patients with TMTV >510 cm^3^ (*P* = 0.0004), CTCs >0.0018 PB cells (*P* = 0.03), or cfDNA >2550 equivalent-genome/mL (*P* = 0.04). Total cfDNA levels and TMTV were independent predictors of outcome. In this experience, ddPCR proved to be promising in the evaluation of multiple compartments in FL, including cfDNA (41).

For the first time in the context of a clinical trial, MRD analysis was assessed by ddPCR in the Relevance protocol (42). At the EOI, 98% and 78% of patients achieved a complete molecular response in the PB and BM, respectively. A complete molecular response was reached more frequently with the rituximab + lenalidomide combination (90%) than with rituximab-chemo (77%) (p = 0.022) (42) **(** **Table 1** **)**.

Mutations other than *BCL2::IGH* are gaining interest for their prognostic relevance in FL, such as the gain-of-function mutations of the *EZH2* gene. Alcaide et al. (43) optimized a multiplex ddPCR for the detection of 4 *EZH2 Y641* and *STAT6* mutations. This assay accurately determined whether the samples harbored either an *EZH2* or a *STAT6* mutation (or both) or whether samples were lacking mutations at both hotspots (43). In a small report, the *EZH2* mutant clone was also detectable in liquid biopsies (44).

These experiences open the way to larger studies to better define the prognostic role of these mutations in FL and if they are suitable markers for MRD.

#### 3.1.1 Other indolent lymphomas

In WM, *MYD88^L265P^* is a diagnostic and predictive biomarker of response to ibrutinib (45). Beside allele-specific RQ-PCR, ddPCR has recently proven to be a suitable and sensitive tool for *MYD88^L265P^* screening and MRD monitoring (46). Both unsorted BM and PB samples can be reliably tested, as well as circulating tumor DNA (ctDNA), which represents an attractive and less invasive alternative to BM for *MYD88^L265P^* detection (46).

*MYD88^L265P^* detection in the cerebrospinal fluid (CSF) by ddPCR is also useful to diagnose the Bing-Neel syndrome (47).

Promising results have been preliminarily shown in splenic marginal zone lymphoma (MZL), where MRD has been assessed in the BM and PB by ddPCR employing IGH allele-specific oligonucleotide (ASO) primers in the phase II BRISMA/IELSG36 trial (48).

### 3.2 Mantle cell lymphoma

MCL is characterized in most cases by a specific t (11, 14)(q13;q32) translocation. It can be detected by FISH in around 70% of MCL at diagnosis and corresponds to the *BCL1::IGH* rearrangement, with BCL1 proliferating activity enhanced by the heavy chain regulatory gene. The most frequent breakpoint is the major translocation cluster (*MTC*) (31, 49, 50). IGH rearrangements are detected by PCR in 80–85% of MCL cases. In at least 10% of cases the detection failure is linked to purely nodal forms without circulating neoplastic cells; *BCL1::IGH* rearrangements are detected by PCR in 30%–40% of such cases, resulting in a proportion of double negative cases ranging from 5 to 10% (51, 52).

The gold standard approach for MRD monitoring relies on *BCL1::IGH* and IGH rearrangements monitored by RQ-PCR, capable of detecting up to 1 clonal cell among 100,000 analyzed (1 × 10^−5^) (52–56). Several large studies sustain the predictive role of MRD in MCL (52–56). Among the most recent, the FIL MCL0208 trial compared maintenance with lenalidomide vs. observation after an intensive chemo-immunotherapeutic regimen and autologous stem cell transplant (ASCT) in 300 young MCL patients (54). A molecular marker (*BCL1::JH* and/or *IGH* rearrangements) was found in 83% of patients, and a MRD negativity was achieved in 78% of patients after high-dose chemotherapy and in 79% after ASCT (54). A time-varying kinetic model, combining the MRD status at two or more consecutive time points (post-ASCT, months +6, +12) was conceived. The combination of the MRD status with the MIPI (Mantle Cell Lymphoma International Prognostic) index proved to be an informative tool in predicting relapse and determining time-to-progression (TTP) (54).

The Nordic Lymphoma Group assessed MRD in 183 MCL patients who underwent an ASCT by performing PCR for *BCL1::JH* and *IGH* rearrangements. Shorter progression-free survival (PFS) and overall survival (OS) were demonstrated for patients who were MRD-positive pre- or after-ASCT: median PFS 20 months in the MRD-positive group vs. 142 months for the MRD-negative patients. OS was 75% at 10 years with a median not reached in the MRD-negative group compared to 35 months in the MRD-positive group (55). This association was even stronger in patients who achieved a complete response (CR) (56).

Also in this setting, the pitfalls of RQ-PCR, especially the contamination risk, the presence of disease levels below the quantitative range and the requirement of a standard curve offer the possibility to improve MRD monitoring by the employment of ddPCR (29, 54).

Drandi et al. (29) compared ddPCR with RQ-PCR in MCL evaluated by both molecular markers. Overall, from a total of 166 patients from four prospective MCL clinical trials, 416 MRD samples were tested by ddPCR, with an over-representation (61%) of below the quantitative range cases by RQ-PCR. ddPCR and RQ-PCR gave comparable results in MRD samples with at least a 0.01% positivity. Amongst 240 samples below the quantitative range with duplicate or triplicate analysis, 39% were positive by ddPCR, 49% negative and only 12% remained positive below quantifiable ddPCR limits. In another experience from the same group, patient-specific IGH rearrangements were amplified and directly sequenced from diagnostic DNA determining specific ASO primers tested both in RQ-PCR and ddPCR. Sixty-seven MCL samples (18 BM and 4 PB diagnostic, and 45 follow-up samples) were tested (28). Only 11.9% were discordant between the two methods, 1 major qualitative discordance and 7 minor qualitative discordances (28).

Della Starza et al. (57) reported alternative targets, such as immunoglobulin kappa-deleting-element (IGK-Kde) rearrangements, as suitable for MRD detection in MCL patients by RQ-PCR and ddPCR. *IGK-Kde* rearrangements were found in 76% (28/37) of cases, representing the sole molecular marker in 73% (8/11) of BCL1::IGH double negative cases. MRD RQ-PCR monitoring was possible in 57% (16/28) of cases, showing a 100% concordance with the conventional targets. Also in this setting, ddPCR showed a good concordance with RQ-PCR (19/24; 79%) and it might help to identify false positive/negative results in samples with low level of residual disease (57).

### 3.3 Diffuse large B-cell lymphoma

Diffuse large B-cell lymphoma (DLBCL) includes a variety of biologic subtypes and variants. The distinction of the cell of origin, i.e. activated B-cell like (ABC) and germinal center B-like (GCB) DLBCLs, is based on the gene expression profile evaluated using the nanostring technology (58). More recently, mutation-based cluster classifications have been provided by the genomic profiling evaluated by NGS (59, 60).

At variance from FL or MCL, circulating cells in DLBCL are rarely detectable, thus many researchers started to use the plasma as a source of tumor DNA, either by extracting cfDNA or the circulating exosomes (61–64). Liquid biopsy of DLBCL at diagnosis and the identification of lymphoma-associated mutations has opened the way to MRD monitoring also in this disease (61, 64). In addition, testing *IGH* and *IGK* clonality on biopsy samples has shown that up to 83% of DLBCL carry an immunoglobulin molecular marker, which can be monitored on ctDNA by NGS and is associated with prognosis and prediction of relapse (62), also in new therapeutic contexts such as chimeric antigen receptor T (CAR-T) cell therapy (65). In this setting, a NGS based approach could overcome some limitations represented by unproductive IGH rearrangements, the variable and generally low amount of cfDNA extracted from plasma and a relapse with a different clone from the baseline one (19, 62).

So far, the application of ddPCR to DLBCL monitoring has been limited to given conditions. One is the monitoring of specific compartments such as the central nervous system (CNS) through analysis of the CSF (66–68). Bobillo et al. (67) characterized tumor tissue mutations by whole exome sequencing in 19 patients with DLBCL (6 restricted CNS lymphomas, 1 systemic and CNS lymphoma, 12 systemic lymphomas). Then, they tested plasma and CSF with a target specific ddPCR designed for each mutation. ctDNA was detectable at diagnosis in the CSF of all patients with primary CNS lymphoma (PCNSL), but not in patients with systemic lymphoma without CNS involvement. At variance, plasma ctDNA was detected in only 2/6 patients with restricted CNS lymphoma with lower variant allele frequencies than CSF ctDNA. CSF ctDNA resulted more sensitive than flow cytometry in documenting residual CNS disease and in 2 cases ctDNA was detected in the CSF months before the full-blown relapse (67).

Also in the experience of Ferreri et al. (68), CSF proved to be a promising compartment to screen and monitor PCNSL in 36 patients at diagnosis and 27 at relapse. A *MYD88* mutation was detectable in 72% of CSF samples by PCR and IL10 messenger RNA in 88% of newly diagnosed PCNSL, never in controls, showing an 82% biopsies-CSF concordance. The high detection rates of *MYD88* mutations in the CSF in PCNSL both at initial diagnosis and at relapse could be further improved by using ddPCR, thus becoming a potential useful tool in patients with lesions unsuitable for biopsy (68).

Another specific condition is the monitoring of expansion and persistence of CAR-T cells in DLBCL patients after infusion. Cheng et al. (69) demonstrated a consistent concordance between flow-cytometry and ddPCR in monitoring anti-CD19 CAR-T cells both *in vitro* and *in vivo*. Similar findings were reported by Monfrini et al. (70) who tested 42 patients (33 DLBCL, 8 primary mediastinal B-cell lymphomas and 1 MCL) treated with commercial anti-CD19 CAR-T cells. A unique ddPCR primer-probe assay was developed to quantify CAR vectors on genomic DNA. CAR-T cells were significantly higher in patients obtaining a CR at 10 days (mean 146 vs 18 CAR+ cells/µl, p <0.05) with major magnitude of expansion at 30 days (mean area under the curve (AUC) 0-30) = 1431.2 vs 584.3; p <0.05). These data were independent from the product employed. ddPCR showed a significant correlation with flow cytometry (r=0.95, p <0.0001 by Pearson correlation) with the advantage of detecting residual CAR-T cells in samples with limited cellularity and/or cryopreserved (bag-leftovers, cryopreserved BM, biopsies, cfDNA) (70). Different assays have been developed for commercial CAR-T cell monitoring. Badbaran et al. (71) designed a single CAR primer/probe combination by sequencing the CAR construct from the lentiviral tisa-cel and axi-cel vectors and designed primers and Black hole quencher (BHQ) probes complementary to the sequences achieving excellent specificity with a detection limit sensitivity of one single CAR copy, corresponding to a sensitivity of approximately 1 in 5000 cells (0.02%) for 100 ng genomic DNA (71).

### 3.4 Chronic lymphocytic leukemia

**Figure 1:**
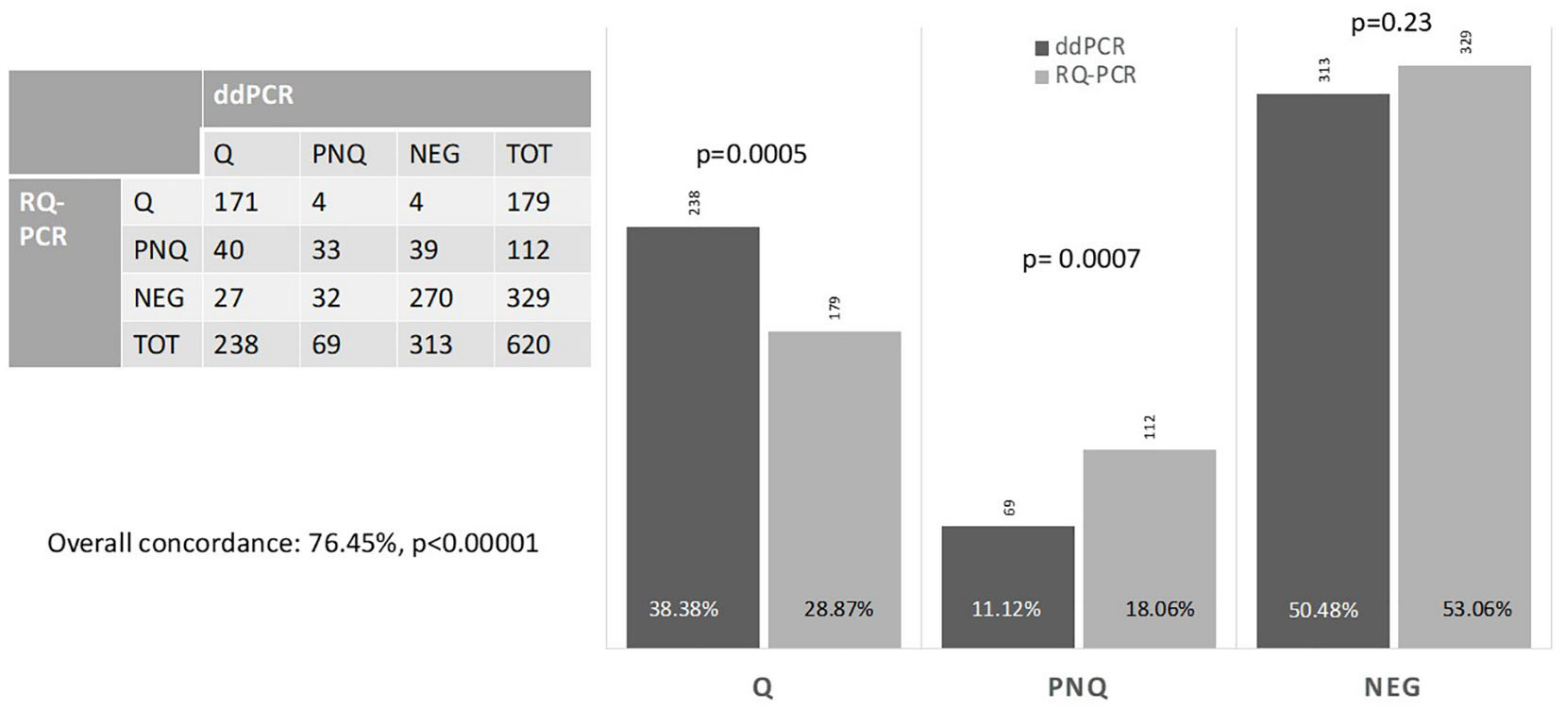
MRD comparison between ddPCR and RQ-PCR. At our Center, we evaluated 216 patients (113 ALL, 47 CLL, 48 FL, 8 MCL) at diagnosis and during the post-treatment follow-up, reaching a total number of 620 evaluations performed by both RQ-PCR and ddPCR, and distributed as follows: 326 ALL, 128 CLL, 142 FL, 24 MCL. The figure shows the overall concordance of the two methods and differences in defining a sample as Quantifiable (Q), Negative (NEG) or Positive Not-Quantifiable (PNQ) for MRD.

Among indolent B-cell malignancies, CLL is the most frequent. The therapeutic landscape of this disease has markedly changed by the availability of targeted drug combinations and the increasing rate of deep CR. MRD monitoring in this context is acquiring progressively increasing importance (2, 72, 73). Standard MRD assessment is based on flow cytometry and on RQ-PCR with IGH ASO primers (73). NGS has also been recently employed as a promising tool that can produce reliable and accurate results in this scenario (74). Data on ddPCR MRD monitoring in CLL are scanty. Our group has conducted a comparative study of ddPCR and RQ-PCR in more than 600 baseline and MRD samples from different lymphoid malignancies, including 128 CLL samples (**Figure 1**) . In all disease entities investigated, a high correlation of the methods was found (76.5%) with most discordances recorded in samples with low RQ-PCR MRD levels, in which ddPCR was able to identify a quantifiable disease more reliably than RQ-PCR (75). In this experience, the advantage of this technique in diminishing the number of PNQ patients was evident (75).

Some experiences have been reported on the monitoring of mutations by ddPCR in CLL. Frazzi et al. (76) tested *TP53* exons 5-6-7 by ddPCR in 47 patients both for mutation and copy number variation. The AUCs for the assays were between 0.91 and 0.98, indicating very high sensitivities and specificities for the deletion assessment with this technique. Concordance between FISH and ddPCR was high for both non-deleted and deleted patients (93.1% and 90.0% respectively). A multiplex approach has been suggested by this experience (76).

Minervini et al. (77) validated a ddPCR based assay for c.7541-7542delCT NOTCH1 mutation. A NOTCH1 mutation was detected in a proportion of CLL cases (53.4%) higher than expected. In follow-up samples, ddPCR showed a statistically significant reduction of the NOTCH1 mutated allelic burden when measured after treatment (median fractional abundance (FA) 11.67% vs 0.09%, respectively, p = 0.01) (77). Hoofd et al. (78) validated a highly sensitive and quantitative ddPCR assay for the NOTCH1 delCT mutation (c.7541_7542delCT). The mutation was detected at allele frequencies as low as 0.024% in 166 CLL tested samples; 25% of unselected cases and 55% of trisomy 12 cases were positive. Association of NOTCH1 delCT and trisomy of chromosome 12 was associated to shorter overall survival (78).

In another experience from our group, mutations and deletions of *BIRC3* were tested by ddPCR in a cohort of 134 CLL with del(11q). *BIRC3* deletion was identified in 105/134 11q- patients (78%) and mutations occurred in 10/134 cases (7.5%), all *BIRC3* deleted, resulting in a biallelic disruption of the gene associated with a poor prognosis. *BIRC3* deletions were identified when carried by 10% of cells (79).

### 3.5 Other chronic lymphoproliferative disorders of B or T-cell lineage

In hairy cell leukemia (HCL), a ddPCR approach has been tested for the molecular detection and monitoring of *BRAF^V600E^* mutation (80–82). ddPCR was retrospectively compared to RQ-PCR in 47 patients (29 HCL and 18 splenic MZL) for the detection of *BRAF^V600E^*. The sensitivity of ddPCR was about half a logarithm superior to that of RQ-PCR (5 × 10^-5^ vs. 2.5 × 10^-4^), with comparable specificity (81). In terms of MRD monitoring, at the end of treatment, among patients in CR, 33% were still MRD-positive by ddPCR versus 28% by RQ-PCR. In another experience, the *BRAF^V600E^* mutational burden has been tested in 35 HCL patients on PB and BM at diagnosis, at the time of response assessment and at relapse (82). Mean values were 12.2%, 0.02% and 16.5% respectively for PB and 23.5%, 0.26% and 13.9% for BM. In 4 out of 6 patients evaluated at response *BRAF^V600E^* was negative in the PB, whilst among patients with long-lasting CR after one course of cladribine the mean *BRAF^V600E^* was 0.05% in 4 cases and negative in 10. These preliminary results suggest that ddPCR may allow to assess the active tumor burden in HCL at different stages of the disease, to refine the response assessment and possibly to identify patients “cured” of their disease.

Limited experience is available regarding the employment of ddPCR in chronic T-cell lymphoproliferative disorders. Tanzima Nuhat et al. (83) reported a good performance of ddPCR in the screening of G17V RHOA mutations in a cohort of 67 patients with peripheral T-cell lymphomas (PTCL), 40 angioimmunoblastic and 27 PTCL-not otherwise specified (NOS), with diagnostic purposes. The ddPCR was compared to NGS: G17V RHOA mutation was detected in 27 of 67 (40.3%) patients by NGS and in 31 of 67 (46.3%) by ddPCR (83). Additionally, variant allele frequencies were highly concordant between the methods (P <.001) (83). Thus, for point mutation detection, ddPCR has a higher sensitivity that NGS, but its targeted nature has to be taken into account, since the whole spectrum of mutations can be missed. In the setting of anaplastic large cell lymphoma, ddPCR seems to be feasible for disease detection and MRD monitoring through ALK fusion transcripts (84, 85).

## 4 Conclusions

Based on the growing body of evidence, ddPCR may be considered as an alternative tool for molecular MRD assessment in lymphoid malignancies. Over the past 5 years, many groups have tested ddPCR for MRD evaluation and several technical advantages have been reported. The main clinical advantage provided by ddPCR is the absolute quantification of the disease, avoiding the need of the diagnostic sample dilution to build the reference standard curve, and the decrease in the number of PNQ samples, that represent a primary unmet need in the clinical practice where treatment decisions are based on MRD monitoring.

Although no guidelines for ddPCR MRD analysis and interpretation have so far been defined, a major standardization effort is underway within ESLHO (European Scientific Foundation for Laboratory Hemato Oncology) through the EuroMRD Consortium (www.euromrd.org) for its future application.

The value of ddPCR for MRD analysis needs to be conclusively documented in the context of prospective clinical trials. This will allow to define whether it could contribute to a further improvement of patients’ management and outcome in different hematological malignancies.

## Author contributions

GA, IDG, and IDS conceived and wrote the paper; MCa, RS, MCo, VB, and MT involved in MRD analysis in acute and chronic lymphoid malignancies; AG and RF revised and conceived the paper. All authors contributed to the article and approved the submitted version.
